# Comparisons Between Frontline Therapy and a Combination of Eltrombopag Plus Immunosuppression Therapy and Human Leukocyte Antigen-Haploidentical Hematopoietic Stem Cell Transplantation in Patients With Severe Aplastic Anemia: A Systematic Review

**DOI:** 10.3389/fonc.2021.614965

**Published:** 2021-04-26

**Authors:** Yuan Yang, Jiang Ji, Zengwei Tang, Bing Han

**Affiliations:** ^1^Department of Hematology, Peking Union Medical College Hospital, Chinese Academy of Medical Science and Peking Union Medical College, Beijing, China; ^2^Department of Hepatobiliary and Pancreatic Surgery, School of Medicine, The First Affiliated Hospital, Zhejiang University, Hangzhou, China

**Keywords:** severe aplastic anemia, eltrombopag, immunosuppression therapy, haploidentical hematopoietic stem cell transplantation, survival

## Abstract

**Background and Aims:** This study aimed at comparing the efficacy and safety of eltrombopag (EPAG) plus immunosuppressive therapies (ISTs) and haploidentical hematopoietic stem cell transplantation (haplo-HSCT) in the frontline treatment for severe aplastic anemia (SAA) patients.

**Methods:** Four electronic databases and Clinicaltrials.gov were comprehensively searched from January 2010 to August 2020. Studies that aimed at evaluating the efficacy and safety of EPAG+IST or haplo-HSCT in SAA patients were included. One-/2-year overall survival (OS), complete response (CR), and overall response rates (ORRs) were indirectly compared between EPAG+IST and haplo-HSCT.

**Results:** A total of 447 patients involved in 10 cohort studies were found to be eligible for this study. A narrative synthesis was performed due to lack of data directly comparing the outcome of EPAG+IST and haplo-HSCT. Consistent with the analysis results in the whole population, subgroup analyses in the age-matched population showed that there was no significant difference in ORR between EPAG+IST and haplo-HSCT groups. However, the CR rate was lower in the EPAG+IST group when compared with the haplo-HSCT group. The incidence rate of clonal evolution/SAA relapse ranged at 8–14 and 19–31% in the EPAG+IST group but not reported in the haplo-HSCT group. The incidence rate for acute graft vs. host disease (aGVHD) and chronic graft vs. host disease (cGVHD) ranged at 52–57 and 12–67%, respectively, for the haplo-HSCT group. The main causes of deaths were infections in the EPAG+IST group, and GVHD and infections in the haplo-HSCT group.

**Conclusion:** EPAG+IST has a comparable ORR and 1-/2-year OS but lower CR rate when indirectly compared with haplo-HSCT in the frontline treatment of patients with SAA. Patients treated with haplo-HSCT may exhibit a high incidence of GVHD, whereas patients treated with EPAG+IST may experience more relapses or clone evolution.

## Introduction

Severe aplastic anemia (SAA) causes severe bleeding, infection, and anemia, which may be fatal. It is mainly caused by immune-mediated destruction of the hematopoietic progenitor cells (1). Currently, human leukocyte antigen (HLA)-matched sibling donor (MSD) hematopoietic stem cell (HSC) transplantation (HSCT) is recommended as the first-line therapy for young adults with SAA. In the absence of matched related donors, immunosuppressive therapy (IST) with antithymocyte globulin (ATG) plus cyclosporine A (CsA) is the recommended first-line therapy (2, 3).

IST with ATG plus CsA is an effective first-line therapeutic option with a 60–80% response rate in SAA patients (3, 4). However, it is associated with the risk of clonal evolution to myelodysplastic syndrome (MDS)/acute myeloid leukemia (AML), hemolytic paroxysmal nocturnal hemoglobinuria (PNH), and relapse during long-term follow-up (5, 6). In addition, approximately one-third of SAA patients remain refractory to IST; this is attributed to the depletion of HSCs in the presence of ongoing immune attack (7, 8).

Transplantation including MSD HSCT, matched unrelated donor (MUD) HSCT, and haploidentical HSCT (haplo-HSCT) is a radically curative option for SAA patients (9, 10). In the absence of MSD or MUD, haploidentical transplantation has been shown to have long survival benefits and acceptable transplantation complications in young SAA patients (11–13). However, it is not widely accepted as a first-line therapeutic option due to high associated risks and a lack of convincing data (14, 15). Our previous study revealed that haplo-HSCT has comparable overall survival (OS) and better failure-free survival (FFS) when compared with IST as the frontline therapy for young patients with SAA, and the long-term OS was the same (16).

Eltrombopag (EPAG) is an oral synthetic small-molecule thrombopoietin receptor agonist that has been found to be an effective option for SAA patients refractory to IST (17, 18). Treatment with EPAG stimulates megakaryocytopoiesis as well as erythropoiesis and myelopoiesis because the thrombopoietin receptor is expressed on both megakaryocytes and HSCs (19–22). Recently, it has been shown that a combination of EPAG and IST exhibits significantly higher rates of hematologic response than IST alone (23, 24).

Haplo-HSCT is widely used in China, probably because of the rapid advances in the transplantation technique and lack of MSD. However, the efficacy and safety of EPAG plus IST have not been compared with those of haplo-HSCT. In this study, we obtained scientific publications on frontline therapy using the two regimens for SAA patients. A systematic review involving 447 patients from 10 studies was finally performed to compare the clinical outcomes and related complications of EPAG+IST and haplo-HSCT.

## Materials and Methods

### Search Strategy

PubMed, Embase, Web of Science, WanFang Database, and Clinicaltrials.gov were comprehensively searched for articles that reported the efficacy and/or safety of EPAG in combination with IST and haplo-HSCT among SAA patients. This search was performed between January 2010 and August 2020. The publication language was restricted to English. The search keywords used were as follows: severe aplastic anemia/SAA, eltrombopag/EPAG/ELT, immunosuppression therapy/IST, HLA-haploidentical hematopoietic stem cell transplantation/haplo-HSCT, survival/prognosis, and progression-free survival/PFS. Moreover, we scrutinized the reference lists of the selected reports to identify additional relevant studies missed in the initial search. Our initial search query was the algorithm of “(((SAA) AND (severe aplastic anemia)) AND (((((eltrombopag) OR (EPAG)) OR (ELT)) OR ((immunosuppression therapy) OR (IST))) OR ((HLA-haploidentical hematopoietic stem cell transplantation) OR (haplo-HSCT)))) AND ((((survival) OR (prognosis)) OR (progression-free survival)) OR (PFS)).”

### Inclusion and Exclusion Criteria

Reports were included if they met the following criteria: (i) patients were diagnosed with SAA/very SAA (VSAA); (ii) patients underwent haplo-HSCT or EPAG plus IST (rabbit/horse ATG+CsA) as the frontline therapy; (iii) reported the OS and/or overall response rate (ORR)/complete response (CR); (iv) described the adverse events, relapse rate, clonal evolution rate, and causes of treatment-related deaths; and (v) published between January 2010 and December 2020.

The exclusion criteria were as follows: (i) animal studies; (ii) review articles or meta-analysis or case reports; (iii) duplicated publications; (iv) non-English papers; (v) studies involving other hematologic malignancies (primary myelofibrosis, non-Hodgkin's lymphoma, chronic myelomonocytic leukemia, chronic myeloid leukemia, acute lymphoblastic leukemia, etc.); (vi) patients with SAA/VSAA refractory to IST; (vii) studies involving salvage HSCT; (viii) studies involving SAA patients treated with IST alone; (ix) studies involving aplastic anemia patients not eligible for the criteria of SAA; and (x) studies without OS, ORR, and CR data.

### Data Extraction

Data extraction was independently performed by two investigators (YY and ZT). In case of discrepancies, they were resolved by consensus between the two investigators. The following variables were extracted: (i) study characteristics (the first author, year of publication, study design and duration, regimen, and number of participants in each study); (ii) patients' basic characteristics (gender, median age, and median follow-up); (iii) the ORR, CR, 1-/2-year OS, incidences of clonal evolution, and disease relapse in patients treated with EPAG combined with IST plus CsA; iv) ORR, 1-/2-year OS rate, the incidences of graft vs. host disease (GVHD), and mortality rates in patients subjected to haplo-HSCT. For the few reports that did not describe the 1-/2-year OS rate, we calculated their OS by using the Engauge Digitizer (Windows version 10.8) software from the Kaplan–Meier survival curve shown in the original articles.

### Statistical Analysis

All the statistical analyses were performed according to the guidelines proposed by the Meta-Analysis of Observational Studies in the Epidemiology group (MOOSE) (25).

Heterogeneity among the included studies was measured using the Q tests and *I*^2^ statistic to assess the extent of the inconsistencies (26). If a probability value of *p* < 0.1 and *I*^2^ > 50%, indicating the existence of significant heterogeneity was found, then a random pooled effect model was performed (27). Statistical heterogeneity was categorized into low (<50%), moderate (51–75%), or high (>75%) according to a predefined criteria (26). *p* ≤ 0.05 was set as the threshold for statistical significance. A funnel plot and Egger's linear regression test was performed to evaluate the potential publication bias for eligible studies using ORR, CR, or OS as endpoints (28). Moreover, a *p* < 0.01 for Egger's test was considered statistically significant. The “Meta” R package was used to perform all pooled analyses. If pooled analysis cannot be performed due to high heterogeneity among included studies or lack of data directly compared the outcomes between the EPAG+IST group and haplo-HSCT group, a narrative synthesis would be performed to indirectly compare the ORR, CR, and OS between the EPAG+IST group and haplo-HSCT group. All statistical analyses were performed using R version 3.6.3.

## Results

### Study Selection

**Figure 1:**
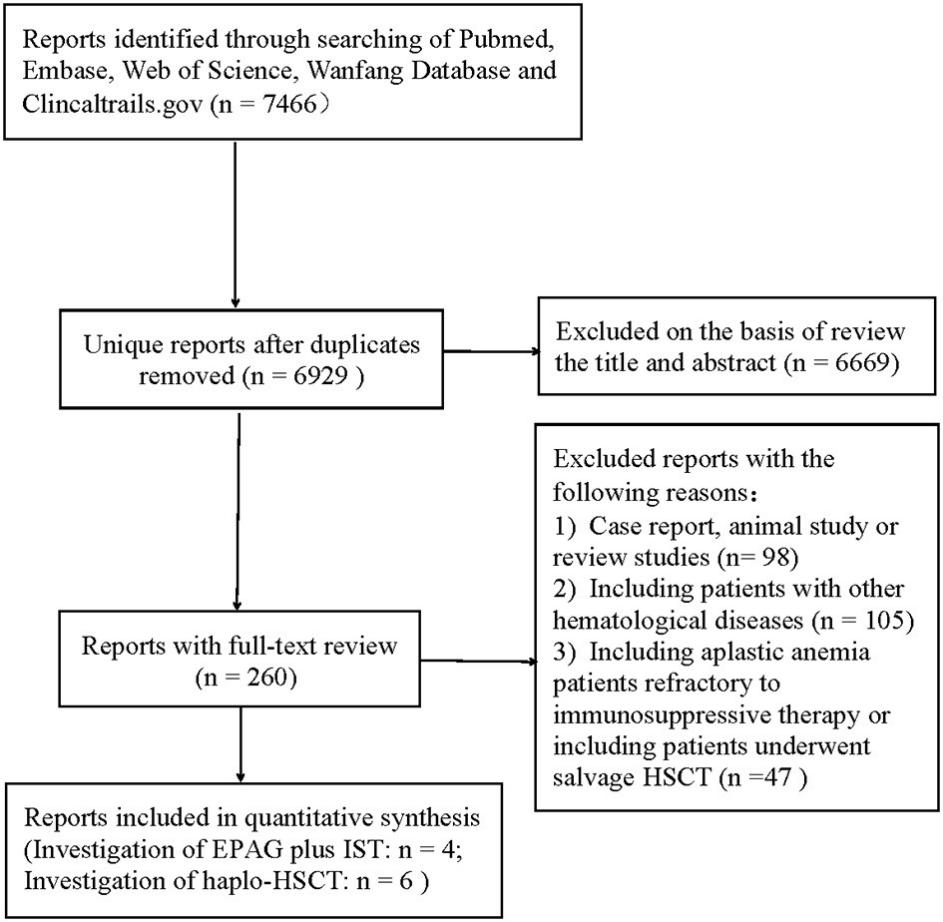
Search flow diagram in our study.

The initial literature search yielded 7,466 articles from the four primary electronic databases. Out of these, 6,669 publications were excluded after reviewing the titles and abstracts, while 260 papers were selected for full-text review. After full-text reviews, 10 articles (3, 12, 14, 23, 29–34) were eligible for this study according to the inclusion and exclusion criteria mentioned above. The screening process was as shown in Figure 1.

### Characteristics of the Enrolled Patients

The selected studies included three prospective and seven retrospective cohort studies. Among them, four studies used EPAG+IST (a total of 252 patients received horse ATG, while 10 patients received rabbit ATG). The other six studies used haplo-HSCT as the frontline therapy; conditioning therapies (predominantly cyclophosphamide+ATG) were used in haplo-HSCT studies. The average median ages of the EPAG+IST group and haplo-HSCT group were 40.6-years (range: 15–60-years) and 9.2-years (range: 8–28-years), respectively (*p* = 0.024). Male patients were 45.6% (range: 30.0–54.3%) in the EPAG+IST group and 61.0% (range: 56.0–70.0%) in the haplo-HSCT group (*p* = 0.036). The incidence rate of VSAA in the haplo-HSCT group was 30.1% (range: 4.3–45.0%); however, there were no data on the incidence rate of VSAA in the EPAG+IST group. The characteristics of eligible studies included in this study are presented in Table 1.

**Table 1:** The characteristics of included studies.

**Group**	**References**	**Disease (no. patients)**	**Study period**	**Study design**	**Male ratio (%)**	**Median age (range), years**	**Study protocol/ conditioning regimen**	**Frontline/salvage**	**Median follow-up (months)**
EPAG+IST	(23)	SAA (92) VSAA (NR)	2012–2015	P	54.3	32 (3–82)	EPAG+horse ATG+CsA	Frontline	24 (2.8–47.4)
	(29)	SAA (21) VSAA (NR)	2012–2018	P	52.4	60 (19–84)	EPAG+horse ATG+CsA+glucocorticoid	Frontline	21 (3–49)
	(30)	SAA (39) VSAA (NR)	2012–2018	R	NR	15 (NR)	EPAG+horse ATG+CsA	Frontline	NR
	(34)	SAA (7) VSAA (NR)	2015–2016	P	30.0	55.5 (39–67)	EPAG+rabbit ATG+CsA	Frontline	88.36 (22.0–104.1)
Average	/	/	/	/	45.57 ± 13.51	40.6 ± 21.04	/	/	44.45 ± 38.05
Haplo-HSCT	(3)	SAA (11) VSAA (9)	2012–2016	R	70	13 (4–18)	CY, ATG, CY, ATG; Flu, Bu	Frontline	29 (1–47)
	(31)	SAA (52) VSAA (24)	2009–2017	R	60.5	28 (18–49)	Bu, CY, ATG	Frontline	24.7 (6.1–103.0)
	(14)	SAA (17) VSAA (11)	2007–2016	R	57.1	8 (2–17)	Bu, CY, ATG	Frontline	38 (9–108)
	(12)	SAA (23)	2007–2015	R	NR	9 (2–17)	Bu, CY, ATG	Frontline	NR
	(33)	SAA (22) VSAA (1)	1998–2012	R	60.9	9.3 (0.6–17.2)	CY, Flu, ATG; BU, TBI, CY	Frontline	NR
	(32)	SAA (18)	2010–2014	R	55.6	8 (3–14)	Flu, CY, ATG	Frontline	24 (3–52)
Average	/	/	/	/	61 ± 5.52	13.1 ± 7.308	/	/	28.93 ± 6.441
*P-*value	/	/	/	/	0.0357	0.0242	/	/	0.2000

*SAA, severe aplastic anemia; OS, overall survival; P, prospective; R, retrospective; NR, not report; ATG, antithymocyte globulin; Elt, eltrombopag; CsA, cyclosporine A; CY, cyclophosphamide; ATG, antithymocyte globulin; Flu, fludarabine; BU, busulfan; TBI, total body irradiation*.

### Indirect Comparison of Overall Response Rate/Complete Response at 6 Months Between the Eltrombopag Plus Immunosuppressive Therapy and Haploidentical Hematopoietic Stem Cell Transplantation Group

Since only 6 month ORR/CR data were available for EPAG+IST, we compared the ORR and CR rates of the two groups.

**Figure 2:**
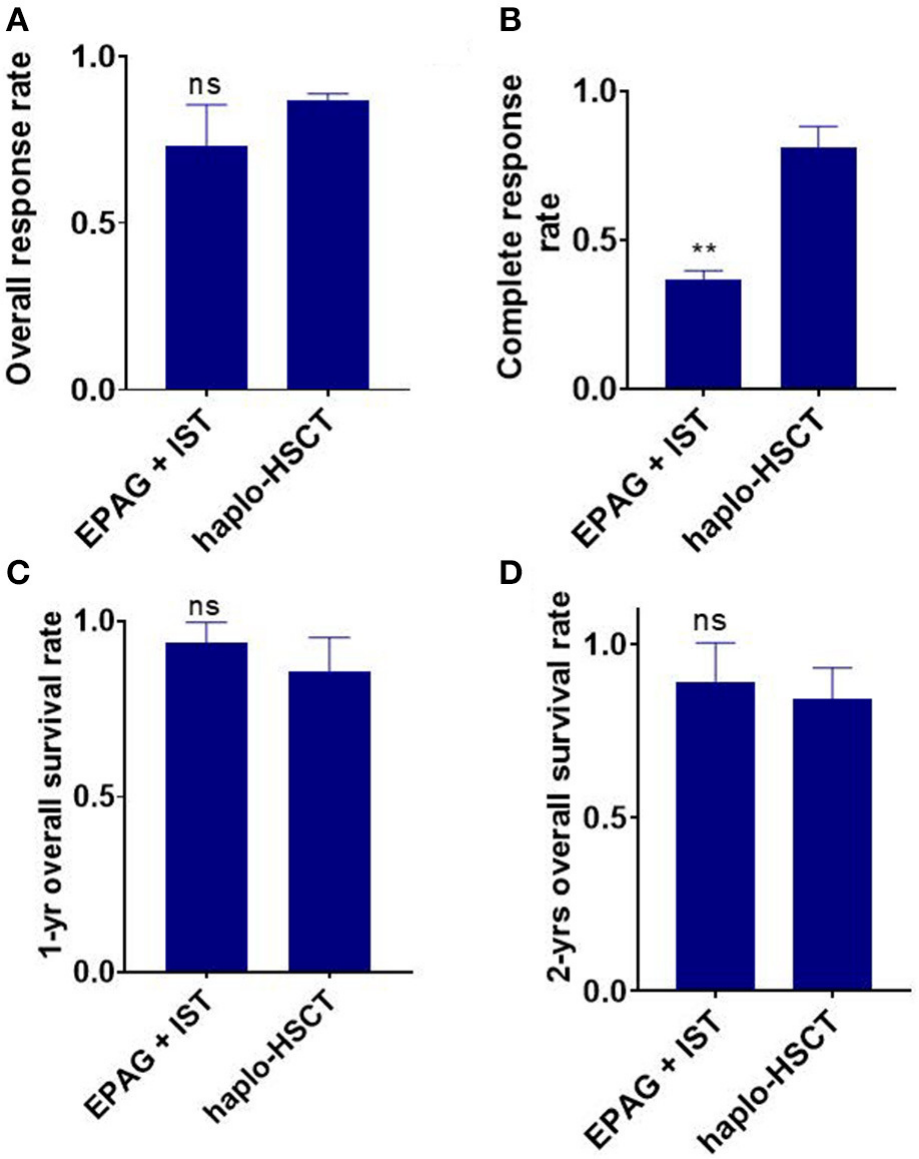
Indirect comparison the overall response rate (ORR), complete response rate (CR), 1-/2-year overall survival (OS) between EPAG+IST and haplo-HSCT group. **(A)** Bar plot shows similar ORR between the two groups. ns, *p* > 0.05, based on the Student *t*-test. **(B)** Bar plot shows significantly lower CR rate in the EPAG+IST group compared with the haplo-HSCT group. **p < 0.01, based on the Student *t*-test. **(C,D)** Bar plots show that the 1-/2-year OS was similar between the indicated groups. ns, *p* > 0.05, based on the Student *t*-test. EPAG, eltrombopag; IST, immunosuppressive therapy; haplo-HSCT, haploidentical hematopoietic stem cell transplantation.

Four eligible studies involving a total of 159 SAA patients in the EPAG+IST group and three studies involving a total of 124 patients in the haplo-HSCT group reported the ORR. The average median age in the EPAG+IST group and haplo-HSCT group was 43.8-years (range: 15–60-years) and 13.0-years (range: 8–28-years), respectively (*p* = 0.024). Male patients were 52.4% (range: 30.0–54.3%) in the EPAG+IST group and 60.5% (range: 57.1–70.0%) in the haplo-HSCT group (*p* = 0.10). The incidence rate of VSAA in the haplo-HSCT group was 39.3% (range: 31.6–45.0%). The ORR of the EPAG+IST group was similar with that in the haplo-HSCT group (*p* = 0.126, Figure 2A).

Two studies involving 115 patients in the EPAG+IST group and four studies involving 142 patients in the haplo-HSCT group reported the CR rate. The average median age in the EPAG+IST group and haplo-HSCT group was 46.0-years (range: 32–60-years) and 10.5-years (range: 8–28-years), respectively (*p* = 0.024). Male patients were 53.4% (range: 52.4–54.3%) in the EPAG+IST group and 58.8% (range: 55.6–70.0%) in the haplo-HSCT group (*p* = 0.13). The incidence rate of VSAA in the haplo-HSCT group was 39.3% (range: 31.6–45.0%). The CR rate was significantly lower in the EPAG+IST group than the haplo-HSCT group (*p* = 0.0012, Figure 2B).

### 1-/2-Year Overall Survival Rate in Eltrombopag Plus Immunosuppressive Therapy and Haploidentical Hematopoietic Stem Cell Transplantation Groups

Two studies involving 113 patients in the EPAG+IST group and six studies involving 188 patients in the haplo-HSCT group reported the 1-/2-year OS. The average median ages in the EPAG+IST and haplo-HSCT groups were 46.0-years (range: 32–60-years) and 9.2-years (range: 8–28-years), respectively (*p* = 0.07). Male patients were 53.4% (range: 52.4–54.3%) in the EPAG+IST group and 60.5% (range: 55.6–70.0%) in the haplo-HSCT group (*p* = 0.10). The incidence of VSAA in the haplo-HSCT group was 35.5% (range: 4.3–45.0%).

The 1-year OS in the EPAG+IST group was similar to that in the haplo-HSCT group (*p* = 0.303, Figure 2C). The 2-year OS in the EPAG+IST group was similar to that in the haplo-HSCT group (*p* = 0.558, Figure 2D).

### Comparison of Deaths and Cause of Mortality

**Figure 3:**
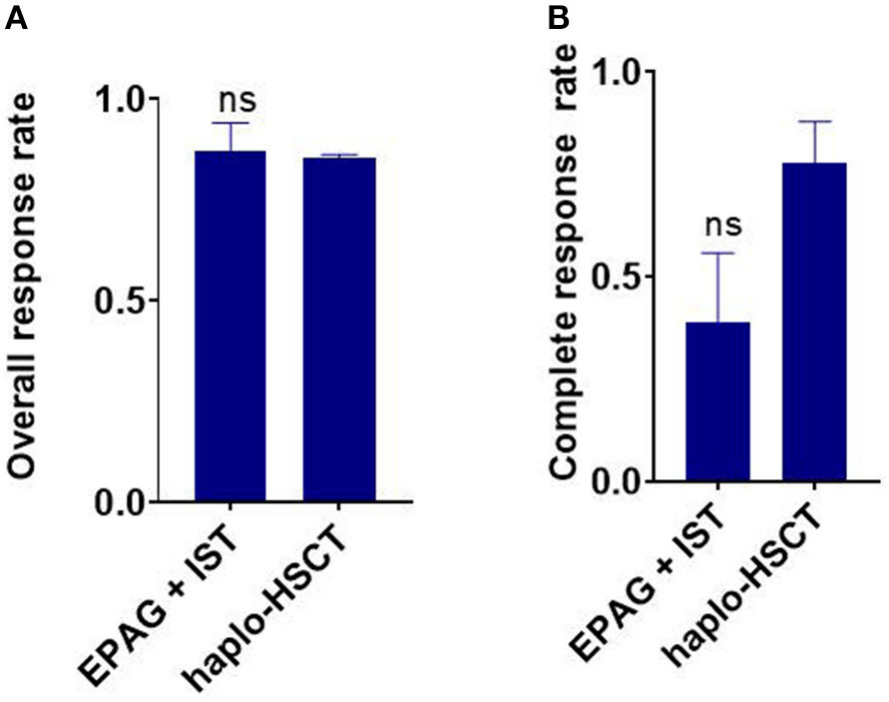
Indirect comparison the overall response rate (ORR) and complete response (CR) rate between EPAG+IST and haplo-HSCT group in age-matched population. **(A)** Bar plots show similar ORR between the two groups. ns, *p* > 0.05, based on the Student *t*-test. **(B)** Bar plots show similar ORR between the two groups. ns, *p* > 0.05, based on the Student *t*-test. EPAG, eltrombopag; IST, immunosuppressive therapy; haplo-HSCT, haploidentical hematopoietic stem cell transplantation.

Two studies involving 113 patients in the EPAG+IST group and five studies involving 165 patients in the haplo-HSCT group reported the causes of deaths. The median age in the EPAG+IST group and haplo-HSCT group was 46.0-years (range: 32–60-years) and 9.3-years (range: 8–28-years), respectively (*p* = 0.07). Male patients were 53.4% (range: 52.4–54.3%) in the EPAG+IST group and 60.5% (range: 55.6–70.0%) in the haplo-HSCT group (*p* = 0.10). The incidence of VSAA in the haplo-HSCT group was 35.5% (range: 4.3–45.0%). The mortality rate in the haplo-HSCT group was similar to that in the EPAG+IST group (*p* = 0.098, Figure 3A).

Two patients died of infections while one patient died of paraneoplastic encephalopathy at 3 months after treatment in the EPAG+IST group. Eight patients died of infections, six patients died of GVHD, four patients died of post-transplant lymphoproliferative disease, two patients died of graft failure, and the remaining two patients died of cardiogenic shock and suicide in the haplo-HSCT group (Table 2).

**Table 2:** Summary of the cause of deaths in EPAG+IST and haplo-HSCT group.

**Group**	**References**	**No. of patients**	**No. of deaths (%)**	**Cause of deaths (no. of deaths)**	**Infection-related deaths (%)**	**GVHD-related deaths (%)**
EPAG+IST	(23)	92	1 (1.1)	Paraneoplastic encephalopathy (1)	–	–
	(29)	21	2 (9.5)	Infections (2)	2 (100)	–
	Total	113	3 (2.7)	–	3 (66.7)	–
Haplo-HSCT	(3)	20	3 (15)	Infection (1), GVHD (1), PTLD (1)	1 (33.3)	1 (33.3)
	(31)	76	11 (14.5)	Infections (3), GVHD (2), PTLD (3), graft failure (2), suicide (1)	3 (27.3)	2 (18.2)
	(14)	28	3 (10.7)	GVHD (1), Not reported (2)	–	1 (33.3)
	(33)	23	2 (8.7)	Cardiogenic shock (2)	–	–
	Zhang (32)	18	6 (33.3)	Infection (4), GVHD (2)	4 (66.7)	2 (23.3)
	Total	165	25 (15)	–	8 (32)	6 (24)

*EPAG, eltrombopag; IST, immunosuppressive therapies; HSCT, hematopoietic stem cell transplantation; GVHD, graft vs. host disease; PTLD, post-transplant lymphoproliferative disease*.

### Clonal Evolution and Relapse Rate in Eltrombopag Plus Immunosuppressive Therapy and Haploidentical Hematopoietic Stem Cell Transplantation Group

Patients in the EPAG+IST group reported the rate of clonal evolution and relapse. Three studies involving 152 patients reported a clonal evolution rate, ranging at 8~14%. The most frequent clonal evolution was loss of chromosome 7. Progression to MDSs or AML was not observed in the studies of Assi et al. or Groarke et al., nor was the development of PNH. Townsley et al. reported that one (1.1%) patient with a complex karyotype progressed to AML, while two (2.2%) patients developed PNH during follow-up. No data were available for clone evolution in the haplo-HSCT group.

Two studies involving 113 patients reported a relapse rate of 19 and 31%, respectively, for the EPAG+IST group. The study by Cheng et al. was the only one that mentioned the relapse rate in the haplo-HSCT group. They documented that there was no relapse at a median of 37.9 months of follow-up. No other relapse was reported for the rest of the studies.

### The Incidence of Graft vs. Host Disease in the Haploidentical Hematopoietic Stem Cell Transplantation Group

Five studies involving a total of 165 patients reported the incidence of acute GVHD (aGVHD) in the haplo-HSCT group. The average median age in the haplo-HSCT group was 9.3-years (range: 8–28-years). Male patients were 60.5% (range: 55.6–70.0%) in the haplo-HSCT group. The incidence rate of VSAA in the haplo-HSCT group was 35.5% (range: 4.3–45.0%). The incidence of aGVHD in the haplo-HSCT was high, ranging at 52–57% (Table 3). The incidence of cGVHD differed considerably in included studies, ranging at 12–67% (Table 3).

**Table 3:** Summary of the incidence of aGVHD and cGVHD in the haplo-HSCT group.

**References**	**No. of patients**	**No. aGVHD (%)**	**No. cGVHD (%)**
Yang et al. (3)	20	11 (55.0)	3 (15.0)
Xu (2018)	76	42 (55.3)	9 (11.8)
Cheng et al. (14)	28	16 (57.1)	8 (28.6)
Choi et al. (33)	23	12 (52.2)	14 (60.9)
Zhang (31)	18	9 (50.0)	12 (66.7)
Total	165	90 (54.5)	46 (27.9)

*HSCT, hematopoietic stem cell transplantation; aGVHD, acute graft vs. host disease; cGVHD, chronic graft vs. host disease*.

Mycophenolate mofetil, CsA, and methotrexate were the main drug for prophylaxis against GVHD and infection, as summarized in Table 4.

**Table 4:** Summary the data on infection prophylaxis and GVHD prophylaxis regimens.

**References**	**Infection prophylaxis**	**GVHD prophylaxis**
Yang et al. (3)	–	CsA, MMF, MTX
Xu (31)	Antibiotic prophylaxis, oral trimethoprim-sulfamethoxazole, fluconazole, acyclovir	CsA, MMF, MTX
Cheng et al. (14)	Non-absorbable oral antibiotics	CsA, MMF, MTX
Choi et al. (33)	Ultrabroad spectrum Antibiotics and antifungal medications	CsA, MTX
Zhang (32)	Ultrabroad spectrum antibiotics and antifungal medications	CsA, MTX, MMF

*MMF, mycophenolate mofetil; MTX, methotrexate; CsA, cyclosporine A*.

The ORR of the EPAG+IST group was also similar to that in the haplo-HSCT group in age-matched population (*p* = 0.793, Figure 3A). The CR rate in the EPAG+IST group was lower than that for the haplo-HSCT group (*p* = 0.064, Figure 3B).

### Subgroup Analyses of 6 Month Overall Response Rate/Complete Response Rate

To make the patients' baselines compatible, we picked those with a similar age [Townsley et al. (23) and Groarke et al. (30) in the EPAG+IST group and Yang et al. (3) and Xu et al. (31) in the haplo-HSCT group]. The median age was 28.5-years (range: 15–39-years) and 20.5-years (range: 13–28-years) in the EPAG+IST and haplo-HSCT groups, respectively (*p* = 0.40). The percentage of males was 55.0% (range: 53.0–55.0%) and 65.1% (range: 60.1–70.0%) in the EPAG+IST and haplo-HSCT groups, respectively (*p* = 0.10). The percentage of VSAA was 34.4% in the haplo-HSCT group.

The ORR of the EPAG+IST group was also similar to that in the haplo-HSCT group in age-matched population (*p* = 0.793, Figure 3A). The CR rate in the EPAG+IST group was lower than that in the haplo-HSCT group (*p* = 0.064, Figure 3B).

### Risk of Bias Among the Included Studies

The items selected for quality assessment of studies included in the EPAG+IST and haplo-HSCT groups are shown in Supplementary Table 1. Overall, two studies showed a low risk of bias, while two studies showed an unclear risk of bias in the EPAG+IST group.

Bias assessment for studies in the haplo-HSCT group showed a high risk of bias in one study and an unclear risk of bias for the other five studies.

## Discussion

Eltrombopag (EPAG), an oral synthetic small-molecule thrombopoietin receptor agonist, was found to be effective for SAA patients that were refractory to either IST or the frontline choice (23). The development of EPAG, with its associated efficacy and safety, has greatly altered the treatment outline for SAA. However, it is associated with relapse and clonal evolution due to its stimulation on both megakaryopoiesis and hematopoiesis of other cell lineages.

Since EPAG has been used for the treatment of AA for only a short time while haplo-HSCT has been widely used in recent years, their long-term effects have not been established. In this study, we searched for all the possible related publications. After careful selection, a total of 447 patients from 10 cohort studies were enrolled. Baseline characteristics showed that patients in the EPAG+IST group were much older than those in the haplo-HSCT group. However, data on disease severity were not available in the EPAG+IST group. When the two groups were compared for ORR/CR, 1-/2-year OS, a few studies had to be excluded due to data absence.

Population characteristic such as age, sex, and disease severity were evenly distributed in the total patient population.

Aged patients usually exhibit poor response to treatment when compared with the younger ones, for either IST or HSCT (35–37). Under this circumstance, we found that EPAG+IST had a very similar ORR (lower in absolute number) than the haplo-HSCT (81% in the EPAG+IST group and 86% in the haplo-HSCT group, *p* = 0.23). Since age was found to be an important factor for therapeutic efficacy, we next performed subgroup analysis for patients with comparable ages. There was no significant difference in ORR between the EPAG+IST group (higher in absolute number) and the haplo-HSCT group (87% vs. 85%). However, there was a low CR rate either in the total population or in the age-matched population in the EPAG+IST group than the haplo-HSCT group, which is comparable with the findings when IST alone and haplo-HSCT were compared (12, 14, 32). As for the OS, the average 1-/2-year OS rate was 94/89% in the EPAG+IST group and 86/84% in the haplo-HSCT group. OS was higher in the EPAG+IST group compared with the haplo-HSCT group.

The mortality rate was relatively small in the EPAG+IST group, and the known causes of deaths were infections and paraneoplastic encephalopathy. In the haplo-HSCT group, the death rate was higher (although not significant), and the main causes of deaths were infections and GVHD. The high mortality rate attributed to GVHD in the haplo-HSCT group implied a relatively high treatment-related toxicity. Moreover, we found that the incidence of GVHD in the haplo-HSCT group was high. Pooled aGVHD and cGVHD were 55 and 33%, respectively. Although most of these GVHD were well-managed and not lethal, they certainly caused a longer hospitalization period, increased medical burden, and reduced the quality of life (38, 39).

Xu et al. (31) reported that donors for adult patients were younger and verified that younger donors might be associated with a lower incidence of GVHD. Furthermore, recent observational studies with small sample size (40, 41) suggested that post-transplant cyclophosphenolate (PTCy) in combination with tacrolimus and mycophenolate is a more effective strategy than PTCy alone in preventing GVHD for older patients with hematological malignancies undergoing reduced-intensity condition (RIC) MUD SCT, but optimal GVHD prophylaxis remains need to be clarified by well-designed randomized controlled trials.

Older patients were found to respond better to EPAG+IST treatment in that they exhibited similar ORR and 1-/2-year OS to those aged younger in the haplo-HSCT group (3, 10, 23). In the age-matched subgroups of EPAG+IST, there were no significant differences in ORR and OS. However, there was a non-significant higher OS and less death rate, probably due to the small number of patients. These findings imply that EPAG+IST has comparable efficacy and OS with haplo-HSCT, even for younger patients, who are the right candidates for haplo-HSCT (35). So far, there was no head-to-head comparison for the frontline treatment of either EPAG+IST with MSD or EPAG+IST with haplo-HSCT. This study elucidates the implications for treatment choice in the era of EPAG. Of course, haplo-HSCT comes with a higher CR rate.

On the other hand, patients in the EPAG+IST group exhibited a clonal evolution rate of 9% and relapse rate of 15%, whereas no relapse or clone evolution was noticed during follow-up (median of 37.9 months) in one study. There was no other clonal evolution/relapse that was reported in the rest of the studies. These findings raised concerns about relapse and clone evolution for EPAG+IST. However, in the age-matched subgroup, in which patients were younger, there was less relapse as well as clonal evolution implying an age-related effect. Although no evidence for the increase of clone evolution rate has been identified when EPAG+IST was compared with the history controls of IST alone as the frontline therapy so far (23, 29), we do see the relapse when EPAG or IST was tapered or withdrawn (36, 42). VSAA patients usually exhibit higher chances of relapse and clonal evolution when compared with SAA patients (43). Therefore, for young VSAA patients, haplo-HSCT is an attractive option when MSD is not available (44), while for young SAA patients, treatment should be balanced depending on the related mortality and the long-term disease outcomes.

There are some limitations for our study. Due to the short period after EPAG approval for AA and the limited use of haplo-HSCT, only a few prospective/retrospective observational cohort studies with small sample sizes were included in this study. Lack of data directly comparing the therapy outcomes between EPAG+IST and haplo-HSCT groups, a narrative synthesis, rather than quantitative synthesis using meta-analysis model was applied in this study to indirectly compare the outcomes between EPAG+IST and haplo-HSCT groups. Moreover, studies on the long-term effectiveness and survival benefits of-EPAG+IST have not yet been published, making the long-term comparison impossible. Only a few of the enrolled studies reported CR/ORR in the haplo-HSCT group. Lack of a VSAA incidence in the EPAG+IST group inhibited comparisons of disease severity. Moreover, differences in treatment and supportive care in different centers, genomic background differences, imbalance of participant baseline characteristics among studies, and different treatment periods may lead to high heterogeneity, making the errors unavoidable.

Furthermore, studies on the long-term effectiveness and survival benefits of EPAG+IST have not yet been published, making the long-term comparison impossible. Only a few of the enrolled studies reported on CR/ORR in the haplo-HSCT group. Lack of a VSAA incidence in the EPAG+IST group inhibited comparisons of disease severity. Moreover, differences in treatment and supportive care in different centers, genomic background differences, imbalance of participant baseline characteristics among studies, and different treatment periods may lead to high heterogeneity, making the errors unavoidable.

In conclusion, this study elucidates the treatment options for SAA, especially in the lack of MSD. Well-designed randomized clinical trials with larger sample sizes and long-term follow-up periods are needed to confirm our findings.

## Data Availability Statement

The original contributions presented in the study are included in the article/Supplementary Material, further inquiries can be directed to the corresponding author/s.

## Author Contributions

YY and BH were responsible for the initial plan and study design, are guarantors and had full access to all of the data, including statistical reports and tables, and take full responsibility for the integrity of the data and the accuracy of the data analysis. YY, ZT, and JJ were responsible for data collection, data extraction, and statistical analyses. YY was responsible for data interpretation and manuscript drafting. All authors contributed to the article and approved the submitted version.

## Conflict of Interest

The authors declare that the research was conducted in the absence of any commercial or financial relationships that could be construed as a potential conflict of interest.

## Abbreviations

SAA: severe aplastic anemia
EPAG: eltrombopag
ISTs: immunosuppressive therapies
haplo-HSCT: haploidentical hematopoietic stem cell transplantation
OS: overall survival
CR: complete response
ORRs: overall response rates
95%CI: 95% confidence intervals
cGVHD: chronic graft vs. host disease
ATG: antithymocyte globulin
MDS: myelodysplastic syndrome
AML: acute myeloid leukemia
MSD: matched sibling donor
MUD: matched unrelated donor
PTCy: post-transplant cyclophosphenolate
RIC: reduced-intensity condition.
