# Supplementary material for: Comparisons Between Frontline Therapy and a Combination of Eltrombopag Plus Immunosuppression Therapy and Human Leukocyte Antigen-Haploidentical Hematopoietic Stem Cell Transplantation in Patients With Severe Aplastic Anemia: A Systematic Review
Source: Front Oncol. 2021 Apr 26;11:614965. doi: 10.3389/fonc.2021.614965 (PMC8107688; doi:10.3389/fonc.2021.614965)
Supplement: Supplementary file 1 [file Table_1.pdf]

Supplementary table 1. Assessment of risk of bias within studies included in EPAG+IST and haplo-HSCT group

Author (Year)	Were adequate eligibility criteria developed and applied	Was the measurement of both exposure and outcome adequate?	Was confounding adequately controlled for?	Was the follow-up complete and adequate in duration?	Are reports of the study free of suggestion of selective outcome reporting?	Was the study free of other problems that put it at a high risk of bias?	Risk of bias
Townsley (2017)	YES	YES	YES	YES	YES	YES	Low
Assi (2018)	YES	YES	YES	YES	YES	YES	Low
Groarke (2019)	YES	YES	Unclear	YES	YES	YES	Unclear
Imada (2020)	YES	YES	YES	YES	YES	Unclear	Unclear
Yang (2019)	YES	YES	Unclear	YES	YES	YES	Unclear
Xu (2019)	YES	YES	Unclear	YES	YES	YES	Unclear
Cheng (2018)	YES	YES	Unclear	YES	YES	YES	Unclear
Xu (2017)	YES	YES	Unclear	YES	YES	YES	Unclear
Choi (2017)	YES	YES	Unclear	YES	YES	YES	Unclear
Zhang (2017)	YES	YES	Unclear	YES	YES	NO	High
